# Impact of multi-source data augmentation on performance of convolutional neural networks for abnormality classification in mammography

**DOI:** 10.3389/fradi.2023.1181190

**Published:** 2023-06-16

**Authors:** InChan Hwang, Hari Trivedi, Beatrice Brown-Mulry, Linglin Zhang, Vineela Nalla, Aimilia Gastounioti, Judy Gichoya, Laleh Seyyed-Kalantari, Imon Banerjee, MinJae Woo

**Affiliations:** ^1^ School of Data Science and Analytics, Kennesaw State University, Kennesaw, GA, United States; ^2^ Department of Radiology, Emory University, Atlanta, GA, United States; ^3^ Department of Information Technology, Kennesaw State University, Kennesaw, GA, United States; ^4^ Mallinckrodt Institute of Radiology, Washington University in St. Louis, St. Louis, MO, United States; ^5^ Department of Electrical Engineering and Computer Science, York University, Toronto, ON, Canada; ^6^ Department of Radiology, Mayo Clinic Arizona, Phoenix, AZ, United States

**Keywords:** mammography, CBIS-DDSM, EMBED, breast cancer, FFDM—full field digital mammography, cancer screening (MeSH), deep learning—artificial intelligence

## Abstract

**Introduction:**

To date, most mammography-related AI models have been trained using either film or digital mammogram datasets with little overlap. We investigated whether or not combining film and digital mammography during training will help or hinder modern models designed for use on digital mammograms.

**Methods:**

To this end, a total of six binary classifiers were trained for comparison. The first three classifiers were trained using images only from Emory Breast Imaging Dataset (EMBED) using ResNet50, ResNet101, and ResNet152 architectures. The next three classifiers were trained using images from EMBED, Curated Breast Imaging Subset of Digital Database for Screening Mammography (CBIS-DDSM), and Digital Database for Screening Mammography (DDSM) datasets. All six models were tested only on digital mammograms from EMBED.

**Results:**

The results showed that performance degradation to the customized ResNet models was statistically significant overall when EMBED dataset was augmented with CBIS-DDSM/DDSM. While the performance degradation was observed in all racial subgroups, some races are subject to more severe performance drop as compared to other races.

**Discussion:**

The degradation may potentially be due to ( 1) a mismatch in features between film-based and digital mammograms ( 2) a mismatch in pathologic and radiological information. In conclusion, use of both film and digital mammography during training may hinder modern models designed for breast cancer screening. Caution is required when combining film-based and digital mammograms or when utilizing pathologic and radiological information simultaneously.

## 1. Introduction

Breast cancer is the most common cancer in women, with 1/8 of women developing breast cancer over their lifespans (1, 2). Screening mammography is a cost-effective and non-invasive method of breast cancer detection. Population-wide mammography screening allows for earlier detection and increased patients’ survival rates (3, 4). However, the workload of the radiologist is high, and the performance of screening mammography is dependent upon the experience of the reader and is prone to high false positives and false negatives (5–7). Because of this, artificial intelligence (AI) models have been developed for breast cancer detection over the past several years with the goal of improving breast cancer screening performance (8–11).

To facilitate model development, many publicly available datasets have been created and released annotated breast cancer images. Digital Database for Screening Mammography (DDSM) is one of the earliest datasets used in computer-aided medical diagnosis systems (CADx) in the 1990s (12). The Curated Breast Imaging Subset of Digital Database for Screening Mammography (CBIS-DDSM) is an enhanced version of DDSM in which lesion specific segmentations were added, and images were converted to more modern formats. CBIS-DDSM has been extensively used to develop numerous AI-powered breast cancer segmentation and classification models (13–18). However, CBIS-DDSM and DDSM contain film-scanned mammography images from the late 1990s, whereas modern mammography is digital. This distinction is important, as digital mammograms have been shown to be superior to film mammograms, offering numerous advantages (19, 20). Recently, more digital mammography datasets have been made publicly available, including the Emory Breast Imaging Dataset (EMBED)—a digital breast mammography dataset with 3.4M mammogram images (21).

To date, most mammography-related AI models have been trained using either film or digital mammogram datasets with little overlap. While many AI models require massive amounts of data before they begin to perform reasonably, there are only a few datasets publicly available—most of them contain film-based images. This has left a gap in knowledge as to the effect of combining these two data types for training, namely whether or not combining film and digital mammography during training will help or hinder modern models designed for use on digital mammograms.

In this paper, we explore the performance of a binary abnormality classification model for screening digital mammograms when trained solely on digital mammograms (EMBED) vs. combined digital and film mammograms (EMBED + DDSM/CBIS-DDSM). To this end, we implemented state-of-the-art binary abnormality classification models trained on different compositions of datasets using full-view screening mammograms. We emphasized the experiment’s reproducibility by providing easy-to-access and annotated source code encompassing the full workflow from research.

## 2. Materials and methods

### 2.1. Dataset descriptions

In this study, three publicly available datasets—CBIS-DDSM, DDSM “Normal” labelled images and EMBED—were utilized for model training and validation with EMBED dataset for solely testing. CBIS-DDSM contains 3103 full mammogram images with lesions annotated as benign or malignant. Normal labelled DDSM mammograms provide 2,778 film mammography images. The publicly released version of EMBED contains 676,008 2D and Digital Breast Tomosynthesis (DBT) screening and diagnostic mammograms for 23,264 patients with a racially balanced data composition. It also contains lesion level imaging descriptors, pathologic outcomes, regions of interest, and patient demographic information. Information for all three datasets is summarized in Table 1.

**Table 1:** Mammogram dataset information.

Dataset	Size	Format	ROI	Selected metadata	Release year	Resolution
CBIS-DDSM/DDSM	5,881 images	Scanned analog film	Lesion segmentations	File location, series description, pathological result	1993	3256 × 1531 –7111 × 5431
EMBED	676,008 images	Digital mammograms	Lesion bounding boxes	Date that the exam was signed, DICOM file path, BIRADS classification, mammogram study description, laterality of pathological result, patient identification number, image laterality	2023	2294 × 1914 –4096 × 3328

**Table 2:** Train, validation, and test dataset configuration.

Dataset	Total images used	Train	Validation	Test	Note
EMBED	2,414	1,441	480	493	Publicly available EMBED dataset with cohorts 1 and 2
EMBED + CBIS-DDSM/DDSM	8,295	4,969	1,656	493	Test set from EMBED dataset only

#### 2.1.1. CBIS-DDSM and DDSM

**Figure 1:**
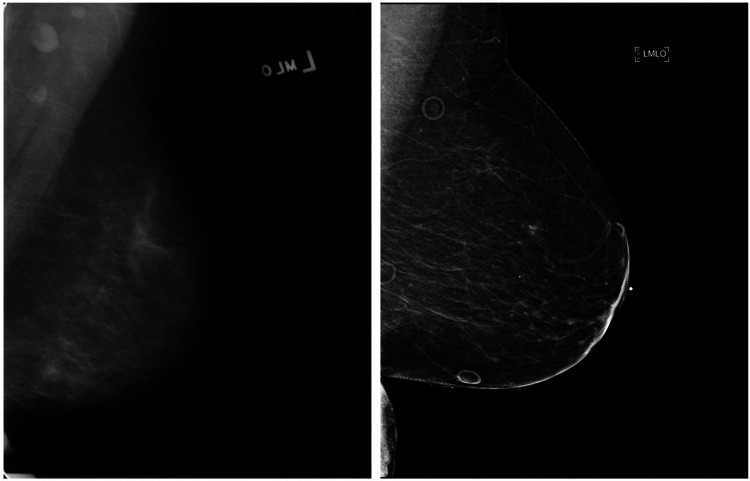
Example of the mediolateral oblique (MLO) view mammograms from the DDSM (**left**) and EMBED (**right**) datasets.

The DDSM dataset contains mammogram examinations on 2,620 patients collected from multiple imaging sites—Massachusetts General Hospital, Wake Forest University School of Medicine, Sacred Heart Hospital, and School of Medicine at Washington University in St. Louis. Images were provided in Lossless Joint Photographic Experts Group image (LJPEG) format with inclusion of both craniocaudal (CC) and mediolateral oblique (MLO) views. However, the LJPEG format has since been deprecated and become difficult for researchers to use (13). CBIS-DDSM was created in 2017 as an augmentation subset of DDSM with images converted to the Digital Imaging and Communications in Medicine (DICOM) format. In addition, specific lesions were segmented and provided in separate files as a mask along with malignant and benign labels. Additional metadata include patient age, tissue density, scanner used to digitize, image resolution, abnormality type (mass or calcification), and performance assessment metrics of CADx methods for mass and classifications. For our study, we obtained negative mammograms from DDSM and abnormal mammographs from CBIS-DDSM. Figure 1 depicts a sample mammogram from the CBIS-DDSM dataset.

**Figure 2:**
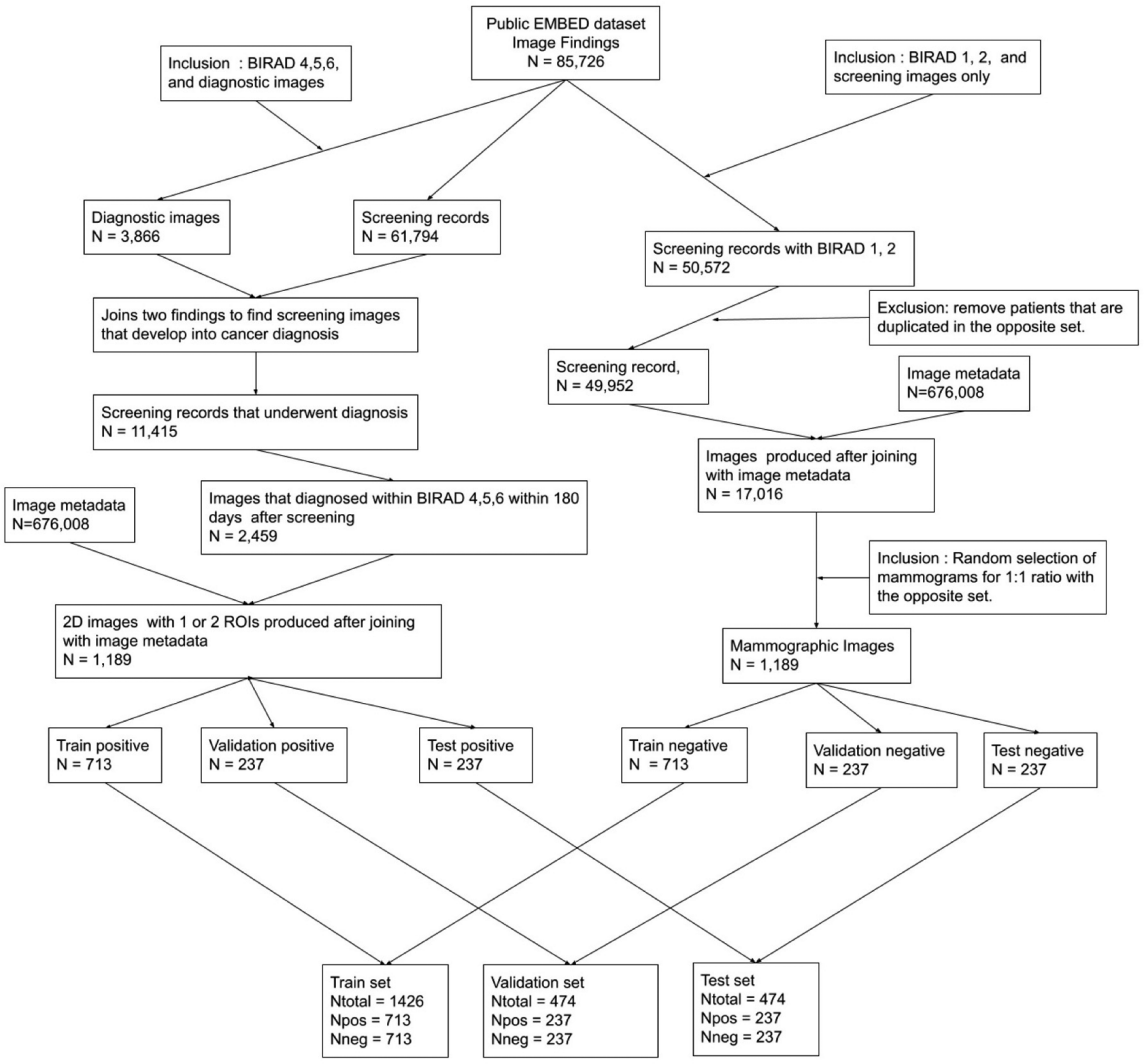
EMBED dataset preparation workflow. Image metadata was joined with image findings to exploit clinical information for each mammogram.

#### 2.1.2. EMBED

The publicly released subset of EMBED contains digital mammograms for 23,264 patients who underwent mammography at Emory University between 2013 and 2020 and includes 364,791 full-field digital mammography (FFDM) images. Whereas other datasets contain patients mostly from a single race, EMBED contains approximately equal numbers of Black (42%) and White (39%) patients. EMBED also contains lesion level bounding boxes for ROIs labeled with imaging descriptors, Breast Imaging-Reporting and Data System (BIRADS) scores, and pathologic outcomes. Figure 1 depicts a sample mammogram from the EMBED dataset.

### 2.2. Data preprocessing and methods

Since the DDSM mammograms are in LJPEG formats, the Stanford PVRG JPEG codec v1.1 was employed to read DDSM images and convert them into 16-bit grayscale PNG images (13). CBIS-DDSM and EMBED images are in DICOM format and were converted into 16-bit grayscale PNG files (22–25). All images were rescaled to 800 × 600 with bicubic interpolation and anti-aliasing to make them fit into 8 GB memory Graphic Processing Units (GPUs) for improved reproducibility. Pixel values were normalized with 16-bit grayscale (26). Images from EMBED were divided into a 60:20:20 ratio for training, validation, and test sets while ensuring that there was no patient leakage between the sets, Table 2 (27–29).

In CBIS-DDSM, ROIs for 3,565 lesions were identified with labels according to their pathology outcomes—benign or malignant. Because all these images contained an abnormality, they were labeled as abnormal (positive class). CBIS-DDSM is a subset of DDSM excluding normal mammograms. Normal mammograms as well as those classified as benign without biopsy (benign-without-callback) were acquired from DDSM and labeled as negatives. From EMBED, screening exams with an original Breast Imaging—Reporting and Data System (BIRADS) score of 0 (i.e., additional evaluation required) were relabeled with the subsequent diagnostic exam BIRADS score. For example, if a patient received a BIRADS 0 on screening mammography followed by a diagnostic exam with BIRADS 4, the final score for the screening study would be BIRADS 4. Conversely, if a patient received a BIRADS 0 on screening mammography followed by a diagnostic mammogram with BIRADS 2, their final score would be BIRADS 2. In this manner, all screening studies with initial or final BIRADS scores of 1 or 2 were labeled as negative, and those with final BIRADS scores of 4, 5, and 6 were labeled as positive. This effectively classified ‘false positive’ BIRADS 0 screening studies without any subsequent abnormality detected in the negative class. BIRADS 3 screening studies were not included in this evaluation as they represent a rare case that is followed more often is subject to a radiologist and institution specific variability. Figure 2 provides an overview of the data preparation procedures employed for the EMBED dataset.

### 2.3. Experimental design and mammogram classifier architecture

Residual Neural Network (ResNet) architectures were chosen for this task as they contain skip connections in convolution layers which minimize residuals between layers, thereby reducing loss rates for classification and allowing the model to learn features at various scale (30–32). It is well documented that ResNet-based models have demonstrated the state-of-the-art performances in breast cancer classification and other cancer classification models (27, 28, 33, 34).

**Figure 3:**
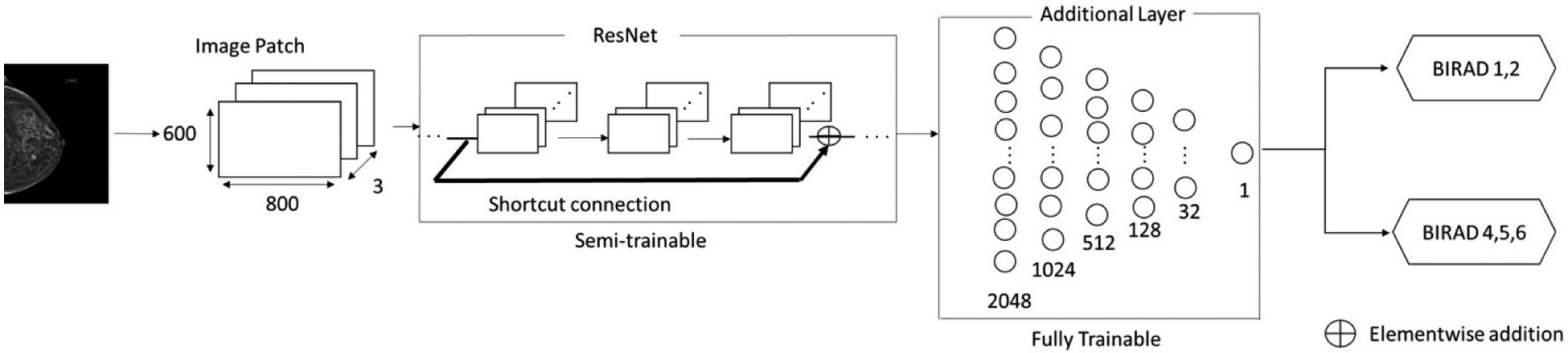
Overview of ResNet-based architecture for abnormality classification. A shortcut connection is shown above as an arrow, Reading input values before layers. Element wise- addition was to minimize residuals produced from each layer to the output values.

Three pretrained ResNet variants—ResNet50, ResNet101, and ResNet152 were selected as feature extractors by freezing some of the weights of the model as semi-trainable layers. The additional six fully connected layers of 2048, 1024, 512, 128, 32, and 1 were added as fully trainable layers followed by the pretrained convolution backbone, Figure 3.

• Input Layer: The input layer has three channels of 800 × 600 pixels to examine signal strengths of cancer. ResNet models take regular three channel RGB colors as inputs. All three channel values in image data are identical because mammogram images consist of grayscale images.
• Activation Function: Rectified Linear Function (ReLu) was selected by the knowledge of previous works and placed in hidden layers. The selected activation function is commonly used in medical image classification tasks as well as general image recognition (35–39).
• Network Configuration: ImageNet pretrained ResNet variations are utilized as semi-trainable base models followed by the additional six fully connected layers, consisting of 2048, 1024, 512, 128, 32, and 1 neuron as a fully trainable layer.
• Avg-Pooling: Average pooling reduces feature map sizes built from convolution layers by dropping the number of parameters and reduces computation overhead (40–42).
• Output Layer: A sigmoid function was selected for the binary classification purpose in the endpoint layer. This function is commonly used in general binary classification and medical image analysis (43–45).

In total, six binary classifiers were trained for comparison. The first three classifiers were trained using images only from EMBED using ResNet50, ResNet101, and ResNet152 architectures. The next three classifiers were trained using images from EMBED, CBIS-DDSM, and DDSM datasets. All six models were tested only on mammograms from EMBED.

In each experiment, the best model was selected based on performance during 50 epochs of training. The selected gradient descent process was the first-order gradient-based optimization of stochastic objective functions (ADAM), guided by previous relevant work in deep learning-powered medical image classifications (46–48). Binary crossentropy was employed for the loss function which has frequently been utilized in medical image analysis (48–50). A range of learning rates was explored with the Bayesian optimization scheme to find the optimal hyperparameters (51–55). All codes used in the experiment are available at https://github.com/minjaewoo/EMBED_Screening_Model.

## 3. Results

The best performing model for EMBED dataset was the customized ResNet50 model, which was trained, validated, and tested using digital mammograms only. The customized ResNet50 model achieved an Area-Under-Curve (AUC) [95% confidence interval] of 0.918 [0.915–0.921], accuracy of 0.918 [0.915–0.921], the precision of 0.907 [0.903–0.911], and recall of 0.929 [0.925–0.933], as summarized in Table 3. To ensure the model performance consistency, bootstrapping over the entire 2,414 images from EMBED was performed by randomly sampling 200 images at a time for 200 times in the testing phase.

**Table 3:** Performance comparisons on different ResNet models before and after multi-source data augmentation.

Dataset	AUC	Accuracy	Precision	Recall	*P*-value*
BIRADS 12 vs. 456 (ResNet50 with EMBED)	0.918 (0.915–0.921)	0.918 (0.915–0.921)	0.907 (0.903–0.911)	0.929 (0.925–0.933)	<0.001
BIRADS 12 vs. 456 (ResNet50 with EMBED and CBIS-DDSM/DDSM)	0.776 (0.772–0.780)	0.776 (0.772–0.780)	0.812 (0.807–0.818)	0.717 (0.712–0.723)	
BIRADS 12 vs. 456 (ResNet101 with EMBED)	0.870 (0.867–0.873)	0.869 (0.867–0.873)	0.908 (0.903–0.912)	0.823 (0.818–0.828)	<0.001
BIRADS 12 vs. 456 (ResNet101 with EMBED and CBIS-DDSM/DDSM)	0.860 (0.858–0.864)	0.861 (0.858–0.864)	0.880 (0.876–0.884)	0.834 (0.829–0.839)	
BIRADS 12 vs. 456 (ResNet152 with EMBED)	0.904 (0.901–0.907)	0.904 (0.901–0.906)	0.879 (0.875–0.883)	0.934 (0.930–0.937)	<0.001
BIRADS 12 vs. 456 (ResNet152 with EMBED and CBIS-DDSM/DDSM)	0.873 (0.870–0.876)	0.874 (0.871–0.877)	0.899 (0.895–0.903)	0.841 (0.836–0.845)	

The numbers in paratheses represent 95% confidence interval calculated using bootstrapping based on random sampling 200 images at a time for 200 times in the testing phase.

* *P*-value was calculated using McNemar’s Test.

The best performing model for EMBED mixed with CBIS-DDSM/DDSM was the customized ResNet152 which was trained and validated with EMBED and CBIS-DDSM/DDSM datasets. This customized ResNet152 model attained an Area-Under-Curve (AUC) [95% confidence interval] of 0.873 [0.870–0.876], the accuracy of 0.874 [0.871–0.877], the precision of 0.899 [0.895–0.903], and recall of 0.841 [0.836–0.845] as depicted in Table 3. Bootstrapping over all 2,414 images from EMEBD was performed by randomly sampling 200 images at a time for 200 times in the testing stage.

The customized ResNet101 did not achieve noteworthy performance in both EMBED and EMBED mixed with CBIS-DDSM/DDSM. In all experiments, performance degradation to the customized ResNet models was statistically significant overall when EMBED dataset was augmented with CBIS-DDSM/DDSM, Table 3. McNemar’s test was deployed for calculating the corresponding statistical significance.

**Figure 4:**
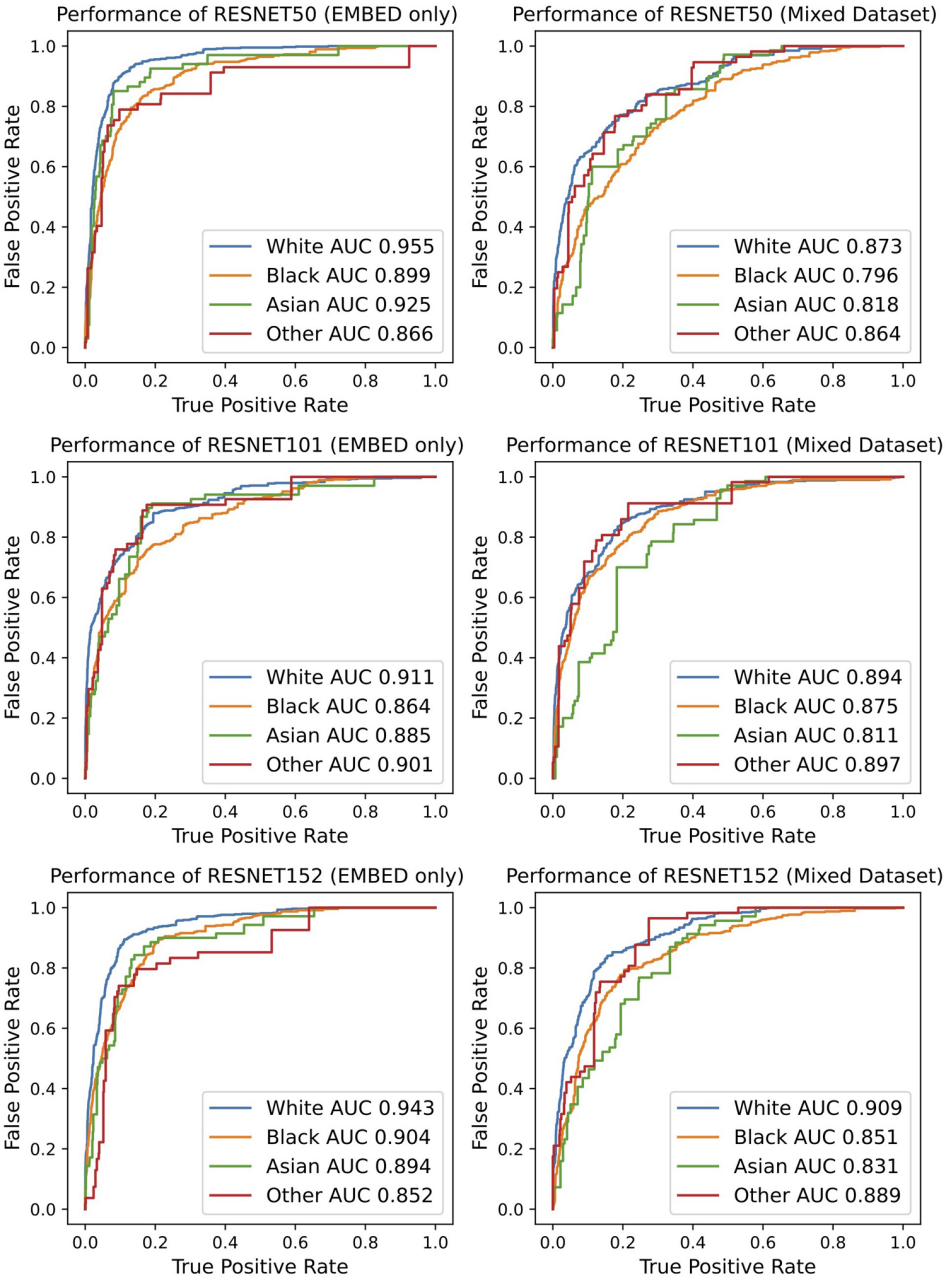
Overview of classification performance stratified by racial subgroups.

The classification performance was further investigated by stratifying the results by racial subgroups, Figure 4. The performance degradation with EMBED dataset augmented with CBIS-DDSM/DDSM was observed in all settings regardless of the racial subgroup. However, it is noteworthy that some races are subject to more severe performance drops as compared to other races. For example, the performance drop presented in AUC ranged from 0.06 to 0.11 in Asian subgroup while the same metric ranged from 0.02 to 0.08 in the White subgroup. Regardless of the degradation effect, the model tended to perform the best in White subgroup. While the ResNet101 did not achieve noteworthy performance throughout the study, it presented the most consistent performance minimally affected by different racial subgroups with the AUC gap smaller than 0.05.

## 4. Discussion

This is the first study, to our knowledge, to evaluate the effect combined film and digital mammography datasets for the development of models to perform abnormality classification in screening mammography. The results indicate that the use of heterogeneous datasets may aggravate the classification performance despite the increase in the size of the training dataset, as compared to the classification model built solely on homogenous datasets.

Specifically, the classification models trained on the heterogeneous dataset with 4,895 film-based and digital mammography images achieved performances ranging from 85% to 87% AUC depending on the choice of the pre-trained weights and structures. Classification models trained on a homogenous dataset with 2,414 digital mammography images achieved performance ranging from 85% to 90% AUC depending on the choice of the pre-trained weights and structures. The degradation of performance was statistically significant when assessed by a non-parametric test for paired nominal data. Furthermore, it was observed that some racial subgroups are subject to more severe performance degradation as compared to other racial subgroups.

Performance degradation when including film-based mammograms can be explained by two factors. First, film-based mammography images may contain different imaging features than digital mammography that, while easily interpretable by a human, appear different from computer vision models. Second, combining mammograms from two datasets can lead to inconsistent labeling which deteriorates the performance of learning algorithms. The commonly used labels for the DDSM are normal, benign, and malignant classes verified by the corresponding pathological information (12, 13). In this study, all biopsied lesions were classified as abnormal regardless of pathology outcome, whereas negative cases were defined as negative screening studies. There was no mechanism in CBIS-DDSM or DDSM to identify cases classified as BIRADS 0 on screening that were subsequently classified as negative. Conversely, the labels for the EMBED were based on radiological information in BIRADS categories; the negatives were defined as BIRADS 1 and 2 on screening or subsequent diagnostic exam, and positives were defined as BIRADS 4, 5, and 6 on the subsequent diagnostic exam within 180 days. It is well documented that there can be a discrepancy between pathologic and radiological findings (56–58). Given that a great number of screening models have been developed using film-based mammograms with pathologic information, our finding poses a serious question on whether those screening models can adequately be adopted in contemporary clinical settings.

The results of this study should be interpreted in consideration of its limitations. First, the composition of the datasets used throughout the study was balanced with respect to the numbers of positive and negative cases. In real-world clinical settings, the number of negative cases far outweighs the number of positive cases in breast cancer screening. The study was designed with an emphasis on its internal validity highlighting the cause-and-effect relationship between multi-source data augmentation and performance degradation. In return, its generalizability to the real-world setting with class imbalanced datasets may be limited. Secondly, the study does not account for the primary source of performance degradation during the multi-source augmentation process. Future studies are warranted to investigate the respective impact of multi-source image features and multi-source label discrepancy on performance degradation. Lastly, some limitations may arise from the specific multi-institutional setting in this study, which involved a film-based mammogram dataset from a single institution and a digital mammogram dataset from a different single institution.

In conclusion, use of both film and digital mammography during training may hinder modern models designed for breast cancer screening. Caution is required when combining film-based and digital mammograms or when utilizing pathologic and radiological information simultaneously.

## Data Availability

Publicly available datasets were analyzed in this study. This data can be found here: https://registry.opendata.aws/emory-breast-imaging-dataset-embed/.
